# Society Meetings

**Published:** 1913-09

**Authors:** 


### SOCIETY MEETINGS.

---

***Brief reports and announcements of meetings of societies of Western New York and of those of general scope are requested from secretaries. Copy should be on hand by the fifteenth of each month. Full transactions will be published at cost of composition.***

---

New York and New England Association of Railway Surgeons. The twenty-third annual session of the New York and New England Association of Railway Surgeons will be held at the Hotel Astor, New York City, on Wednesday, October 22d, 1913. A very interesting and attractive program has been arranged. Dr. Hugh H. Young of Baltimore will deliver the

"Address in Surgery." Railway surgeons, attorneys and officials and all members of the medical profession are cordially invited to attend. Dr. John W. LeSeur, President, Batavia, N. Y.; Dr. George Chaffee, Corresponding Secretary, 338 47th street, Brooklyn, N. Y.

---

AMERICAN PROCTOLOGIC SOCIETY. Fifteenth annual meeting, Minneapolis, June 16 and 17, 1913. President, Dr. Louis J. Hirschman, Detroit. Officers elected for the ensuing year: President, Joseph M. Mathews, M. D., Louisville, Ky.; vice-President, Jas. A. MacMillan, M. D., Detroit, Mich.; Secretary-Treasurer, Alfred J. Zobel, M. D., San Francisco, Cal.

The following is an abstract of the principal papers read:

"A Method of Operating on Fistula Without Cutting Muscular Tissue," by Rollin H. Barnes, M. D., St. Louis. This method is used in cases involving the sphincter muscles. An incision is made external to the sphincter, similar to that made when incising an ischio-rectal abscess. Through this opening the scar tissue is dissected out up to the internal opening. An incision is then made at the skin margin so that the middle of this incision passes through an imaginary longitudinal line drawn from the internal opening. A submucous dissection is then channeled out up to the internal opening. Gauze drainage is kept in this until the external wound is healed sufficiently. Then the submucous tract, which remains, is incised under local anesthesia. No muscular tissue having been cut, the function of the sphincters is preserved intact.

Report of a case of Fecal Tumor associated with Hirschsprung's Disease, by Alois B. Graham, A. M., M. D., Indianapolis. A French woman, aged 27, stated that she had undergone three abdominal operations for Megacolon. Present illness dates from birth. Not unusual to go a week or ten days without a stool, and then evacuation was produced only by means of enemata. At the age of 12 her condition was diagnosed as one of pregnancy on account of the vomiting and the appearance of the abdomen. At 19 she suffered complete intestinal obstruction. A large fecal tumor was removed from the sigmoid. Six months later she was operated for post-operative adhesions. No resection of the bowel or short-circuiting operation was performed. At 25 she suffered an attack of complete intestinal obstruction, and a large fecal tumor was removed. Patient stated that the bowel was plicated in closing. Wound healed promptly, but she remained in the hospital for three months purely for clinical purposes.

August, 1912, she, for the third time, presented symptoms of complete intestinal obstruction. She had been absolutely constipated for seven days. Abdomen enlarged and everywhere tympanitic except in the lower right quadrant, where there was a dull area corresponding to a large tumor which could be readily palpated. Tumor, a fecal mass, was exceedingly hard and did not pit on pressure. It could be easily moved in every direction throughout the abdomen. Attacks of violent, colicky pains were frequent. Vomiting was persistent, pulse 120, Temp. 101 F. Hydrogen Peroxide, introduced into the rectum, had no effect on the tumor, but produced excruciating pains over the entire abdomen. Patient consented to operation with the promise exacted that nothing radical be attempted. She requested that the fecal tumor be removed, but refused to give her consent to any short-circuiting or resection of the bowel. Median incision. No adhesions. Fecal tumor in sigmoid. Tumor of "stony" hardness. Its greatest circumference was 19¾ inches, its weight was 64 ounces. The dilatation which was confined to the sigmoid was very marked, the greatest circumference being 20 inches. Uneventful operative recovery, discharged on the tenth day. She gained in weight and appeared to be in the best of health. She experienced no difficulty in procuring daily evacuations with the aid of small doses of cascara.

December 15th, 1912, was the date of her last visit to the writer's office. At this time she was doing nicely. Information was received the latter part of April that patient had gone to Chicago from Indianapolis. She evidently suffered another attack of intestinal obstruction. She was operated there April 19th, 1913, and died three days later.

"A Further Consideration of Sir Charles Ball's Operation For Internal Hemorrhoids," by Alfred J. Zobel, M. D., San Francisco.

After a trial of this operation the author sums up as follows: As a modification of the old ligature operation, it is better than the latter, and at the same time is far superior to the clamp and cautery operation, in that it takes care of and avoids the recurrence of that revoluted anal skin ring which generally becomes markedly edematous immediately after these operations, leaving behind skin tags after the swelling subsides. In every instance in which the essentials of Ball's technique have been followed out carefully the author's results have been exceedingly satisfactory.

"Deductions Based on an Analysis of 3000 Rectal Cases," by T. Chittenden Hill, M. D., Boston. There was a total of 1120

operations. It was found that rectal ailments were more common among males, the ratio being three to two.

Hemorrhoids formed, 41 per cent. of the total; abscesses and fistulæ, 18 per cent.; pruritus ani, 8 per cent; anal fissure, 10 per cent.; Colitis, 6 per cent.; prolapsus ani and procidentia recti, 3.7 per cent.; cancer of the rectum and sigmoid, 2 per cent.; benign growths, 1.5 per cent.; stricture, 1.5 per cent.; Syphilis, 2 per cent.; constipation, 2.8 per cent.

Other miscellaneous conditions were recorded which made up but a fraction of one per cent.. such as anal verrucca, congenital stenosis, petulous anus, pilo-nidal sinus, furuncles, foreign body, incontinence, coccygodynia, trauma, sigmoid diverticulitis, etc.

"Z-Plastic Operation For Anal Stricture," by Wm. M. Beach, M. D., Pittsburgh. Extensive cicatrices, resulting from trauma, and involving the partial or entire anal circumference, not infrequently resist the usual methods employed to restore the physiologic function of the anus. He therefore employed what he terms a Z-plastic method when operating on an anal stricture. The principle underlying the procedure is the transposition of dermic tissue in such manner as to obliterate the crest of the fibrous band. The first incision is made along the crest of such a band; then incisions are made at right angles from both ends, but running in opposite directions, thus approximating the letter Z. The flaps thus outlined are dissected up, transposed and sutured. Various modifications are permissible, according to the extent of the stricture.

"Sphincteric Atrophy—Causes, Consequences and Treatment," by Ralph W. Jackson, M. D., of Fall River. Muscular atrophy about the anus produces more serious consequences than hypertrophy. The physiology of defecation is studied, and the action of the internal sphincter and of the external sphincter and levators sharply contrasted with their different innervation. This is preparatory to consideration and classification of the causes of sphincteric disuse and consequent degeneration. Congenital causes are found in imperforate anus and congenital ano-vaginal cloaca. Coincidental with general weakness cases occur in infants, the aged, and the extremely ill. Traumatic causes are faults of proctologic operations and after care, or obstetric lacerations, or due to prolonged divulsion by protruding piles or procidentia. Nerve causes are primarily sympathetic as in rectal stenosis, or central as in spinal cord lesions.

Degeneration or absence of one sphincter without impairment of the other is considered.

"Further Observations on the Surgical Anatomy of the Large Bowel," Granville S. Hanes, M. D., Louisville. Few realize that the capacious portion of the colon is at its cecal extremity. The diameter of the average cecum is estimated at three inches, which is about the same as the rectum, though the cecum and ascending colon have a much greater capacity than the rectum and lower extremity of the sigmoid. The large intestine gradually decreases in size from the cecum to the rectum; the descending colon measuring one and one-half inches, or even less, at its narrowest point. These physical conditions explain in a measure the locality to which large quantities of fluids are transported when injected into the rectum.

The question of antiperistalsis in the large intestine in man is yet to be settled. It has been suggested that anastalsis may be inferred to exist in the proximal human colon for the reason that rectal enemas have been observed to traverse the entire length of the colon and escape through an artificial opening in the cecum. Also, because surgeons have attempted to stop a fecal-fistula discharge by transplanting the ileum into the transverse colon and sigmoid, but without success. The fact that rectal enemas have been seen to pass through the cecal fistula is, he is confident, little evidence of the operation of an antiperistaltic force.

An ordinary colon tube was introduced two or three inches into the rectum of a dog, and through a funnel inserted into the proximal end of the tube was poured in bismuth-buttermilk, and by the X-ray the author observed it traverse the large intestine to the ileo-cecal junction with no sign of antiperistaltic movements. Similar experiments were made on children with corroborating observations. He has seen a pint of bismuth in suspension, when introduced into the rectum of an adult, pass around to the cecum in a few minutes with no evidence of aid by anastalsis.

Under normal conditions peristalsis in the large bowel is a slow process, and it is no more than natural to suppose that anastalsis is also slow in its operation. The brief time, then, required for fluids to pass from the rectum to the cecum compels us to consider the influence of other and more potent agents on the intestinal contents. Two factors are in operation when fluids are conveyed from the rectum to the cecum. The first is the distensible and elastic nature of the intestinal tube; and the second is the hydraulic principle which controls fluids wherever they may be. If fluid is forced rapidly into the rectum that organ will be seen to be widely distended; but this same fluid can be seen to make its way up the intestinal tube along the path of least resistance. The distended rectum, because of its elastic nature, presses upon the contents till every drop of fluid within its lumen is subjected to an equal pressure. So if additional fluid is forced into the rectum the same factors will continue to operate.

If the ileum is transplanted into the transverse colon or sigmoid the watery intestinal contents will be forced by the elastic intestinal tube in the direction of least resistance. The right segment of the colon is the capacious portion of the large bowel, so if fluids are under greater intestinal pressure in the lower bowel the fluid contents will travel up to the cecum.

The author says, that even if we do admit the existence of anastalsis in normal conditions of the colon, he does not believe it to be an important factor in conveying fluids from the rectum up into the colon.

Hanes had a series of three X-ray pictures made on the same individual to show what actually happens when tubes are introduced into the bowel. The first shows a thirteen inch proctoscope introduced its entire length. The distal end is one inch above the umbilicus. The second shows an ordinary colon tube introduced its full length after the removal of the proctoscope. The tube passed along the sigmoid up to the highest point (one inch above the umbilicus), and then turned upon itself, the distal end passing back to the rectum. The third radiograph shows the bowel injected with bismuth-buttermilk, and the thirteen inch sigmoidscope introduced again. This picture shows that it is impossible to pass any instrument high up in a normal colon, except by the greatest accident. The sigmoid is lifted up into the abdominal cavity; its lower arm is occupied by bismuth and the metal tube; while the upper segment of the sigmoid is seen very distinctly where it has dropped back from a point opposite the umbilicus into the pelvis to its junction with the lower extremity of the colon. He claims the latter radiograph proves that it is impossible to pass a non-flexible instrument beyond the first half of the sigmoid.

To control the outflow of fecal material in colostomies the author has found, in five cases operated since January of this year, that the hard rubber rod can be allowed to remain permanently, when used as in the Maydl operation. The opening in the intestine is above the rod. A thin gauze dressing is applied over the bowel, and a strip of gauze is thrown around the knuckle of the intestine and overlying gauze is then tied under the supporting rod. The strip of gauze constricts both the upper and lower segments of the bowel, and exerts a most satisfactory control over these artificial openings.

"The Ano-Rectal Line: Its Clinical Significance," by Collier F. Martin, M. D., Philadelphia. After discussing the development of the anus and rectum, Martin states that the ano rectal line, or dentate border, has a very important clinical significance, in that it is the point at which both the blood supply and the nerve supply become differentiated. Above it the blood is carried by the portal circulation to the liver; while below it the blood stream mingles with the general circulation by way of the inferior vena cava. Above it the rectum is supplied only with visceral or sympathetic nerve fibers, which below it, the anus and its surrounding structures are supplied with spinal nerves, and by sympathetic filaments. These spinal nerves carry sensory impulses common to nerves having specialized cutaneous nerve-endings.

Below the ano rectal line, as evidence of irritation of the spinal innervation, sensory disturbances are expressed in terms of pain, itching, formication, and in alterations in spinal sense of touch and temperature, with their modifications such as dryness and moisture. Stimuli producing these sensory disturbances show their presence by exciting motor contraction, or by inducing alterations in secretion.

Above the ano rectal line all of the specialized spinal sensations are absent, only the visceral sensations being present. In the rectum it is only pressure and muscle-sense that appeal to our consciousness. This sensation is translated in the brain into a desire for stool, which desire is inhibited or assisted voluntarily, as occasion may require.

Excessive spasm of the involuntary muscles supplied by visceral nerves produces an unpleasant sensation, which differs from pain of spinal origin in that it is difficult to localize, and may be described more as an ache, which is difficult to bear and exhausting to the patient.

Lesions of the crypts of Morgagni, since they involve both the visceral nerve supply of the rectum and the spinal innervation of the anus, are associated with many disturbances of the reflexes.

Infection and malignant processes, occuring above the dentate border, tend to spread upwards, by way of the deep lymphatics, to the pelvic or uro-genital organs, or to the liver, via the portal system. Below the ano rectal line superficial abscesses result from infections of the proctodeum and the rectal crypts. Malignancy here is associated frequently with extension to the inguinal glands.

In general there is a marked tendency for pathologic processes to limit their invasion to the embryonic structure in which they began; the ano rectal line being the "great divide" between the ectodermic and the entodermic structures. Rectal infection and malignancy rarely extend below the dentate border, while anal pathology usually remains below this line and the levator ani muscles.

Ano rectal symptomatology is equally differentiated. The subjective symptoms of a pathologic process bear little relationship to the lesion, per se, but depend upon the interference with the functions of the spinal or sympathetic nerve supply of the tissues involved, whether this interference be mechanical, inflammatory or functional.

"Further Observations on Pruritus Ani: Its Probable Etiologic Factor; Results of Treatment," by Dwight H. Murray, M. D., Syracuse. He found no reason for materially modifying his former reports, but has gathered data which helped to prove their correctness. He found streptococcic infection in three cases of pruritus ani and vulvae, and in four cases of pruritus that had involved the scrotum as well as the anus. These complicated cases improved, with the exception of two vulva cases, by the use of the vaccine treatment.

During the past year Dr. Murray has increased his former series of thirty-two cases by twenty-five additional cases, in five of which streptococcic infection was not found. These cases showed other infections, which still further proves the cocigenous nature of pruritus ani; and also demonstrates that other bacteria than streptococci may bear a casual relationship, as was hinted in his first paper on this subject.

His cases, so far as he has been able to determine, have not been affected by diet. Since Dr. Murray discovered the infection in pruritus ani he has never interfered with the food of any patient; neither has he restricted them in the smoking or drinking habits. The improvement under the vaccine treatment, without regard to eating, drinking or smoking, gives him additional proof for the bacterial theory.

During the past year he has carefully investigated as to whether or not the itching extends into the anal canal beyond Hilton's white line, with the result that only in one instance did it extend beyond that point, and then only for a short distance.

His investigations of the past year have given him additional proof that pruritus ani is not caused by any local lesion within the anal canal, and that when such lesions exist with pruritus ani they are coincidental.

In the cases that have been operated for local lesions the pruritus ani has not been permanently improved as a result of the operative procedure.

He said that rectal and general surgeons have observed many cases of fistulæ with discharges upon the anal skin, without pruritus ani being present. The same is true of hemorrhoids, constipation and other rectal lesions, pruritus ani occurring in only a small proportion of such cases. He, therefore, still holds that when pruritus ani exists in connection with other lesions that it is a coincidence. In his 1912 report he gave a summary of nine hundred consecutive rectal cases wherein this fact was established fairly well.

He referred to the opsonic index, or more properly the coefficient of extinction of opsonins, and claimed that much valuable information was to be gained by this test.

His work shows that if a complicating infection exists, and other bacteria than streptococci are found to be the sole invading organisms, we must use the corresponding autogenous vaccine. The opsonic index, following a bacterial diagnosis, is the proper method for determining this.

The results of treatment and the history of patients prove to him that if pruritus ani exists with local lesions which demand operation, that the prognosis depends upon whether a skin infection is present or not. If the skin infection is present the local lesions may be cured by the operation, but the patient should not be led to believe that the pruritus ani will also be cured by it. Per contra, if a skin infection does not exist with a local lesion and itching, the prognosis may be that the itching will very likely cease with the cure of the local lesion.

After personal investigation in treating, watching results, noting how cause, effect and results dovetail together; comparing these investigations with statements and theories made in text books, and in articles appearing from time to time in medical journals, and containing no definite pathology or scientific reasons for cause and effect; Murray cannot understand how the profession will uphold such theories, rather than the bacterial theory which has been so well proven in his own cases and confirmed by other observers.

The uniformity of the bacteriologic findings is a strong support for the bacterial theory of the etiology of pruritus ani. The chronicity of all the cases, the uniform symptoms, the similar conditions of the skin, the locality, the regularity as to the time of attacks, the uniformity of itching outside of Hilton's white line, the uniform blood findings as to the coefficient of extinction of opsonins, and the fact that all local applications which have given beneficial results in the past have contained a strong germicide, all point directly to a common cause. Further confirmation is found in the uniformly good results of treatment with autogenous vaccine of the variety of bacteria against which the patient has a low phagocytic power, and in the lack of good results by the various haphazard methods of treatment in general vogue.

His reference to fissures in previous papers having been misunderstood by some, he desired to state that he had referred only to fissure-like cracks of the skin, and not to anal fissures or ulcers.

Endo's medium is used to plate the cultures. The vaccine employed is of the strength of one billion to the CC., beginning with two minims, or one hundred and thirty millions.

Dr. Murray refers to a paper written by Dr. Jerome Wagner of New York City, published in the May number of the Medical Review of Reviews, in which Dr. Wagner reports some erroneous ideas claimed to have been gleaned from reading Murray's first two reports. Dr. Wagner not having been able to confirm these reports, Dr. Murray pointed out the errors of technique in Dr. Wagner's work, as well as his errors in the interpretation of the reports.

Dr. Murray gave statistics in favor of his theory, drawn from three years' original work on the subject; he also gave a summary of the results of treatment, showing the favorable clinical results with autogenous vaccines in a large majority of the cases treated.

He summed up his conclusions as follows:

1st. Results of the past year's work continue to uphold the correctness of the bacterial theory of pruritus ani.

2d. It is advisable to make a bacteriologic examination of all cases of pruritus vulvae; also of cases of scrotal pruritus.

3d. The coefficient of extinction of opsonins is a valuable aid in diagnosis in complicated and obstinate cases.

4th. Pruritus ani in this series of cases rarely extends above the white line of Hilton, and it is still subjudice.

5th. The presence of a skin infection with a local lesion begets an unfavorable prognosis for the cure of pruritus ani by an operative procedure.

6th. The absence of a demonstrable skin infection and the presence of local lesion, with pruritus ani, will justify us in making a favorable prognosis for the cure of the pruritus ani by an operative procedure.

7th. Pruritus ani, with such infection as we have demonstrated, and a lesion existing in the anus or rectum, according to his statistics, is a coincidence, and the latter lesion is not the cause of the pruritus ani.

8th. The sphincter muscle does not allow a leakage of rectal mucous upon the anal skin of one who has pruritus ani, except there is a patulous anus, any more than it does in a normal individual who has no pruritus ani. The moisture of the parts is due to a low grade inflammation of the infected anal skin.

"Treatment of Fistula-in-Ano," by J. A. MacMillan, M. D., Detroit. There are three essentials for the operation for this condition:

1st. An incision that will open up every ramification of the fistulous tract.

2d. The excision of the fibrous tissue which forms its walls.

3d. Free drainage and a regulation of the granulation by means of pressure by gauze packing.

